# Clinical rationale for dietary lutein supplementation in long COVID and mRNA vaccine injury syndromes

**DOI:** 10.12688/f1000research.143517.3

**Published:** 2024-10-17

**Authors:** Anthony M Kyriakopoulos, Greg Nigh, Peter A McCullough, Stephanie Seneff

**Affiliations:** 1 Department of Research and Development, Nasco AD Biotechnology Laboratory, Piraeus, Greece; 2 Naturopathic Oncologist, Immersion Health, Portland, Oregon, USA; 3 McCullough Foundation, Dallas, Texas, USA; 4 Computer Science and Artificial Intelligence Laboratory, Massachusetts Institute of Technology, Cambridge, Massachusetts, USA

**Keywords:** Lutein; chronic illnesses; post-COVID and mRNA vaccination injury syndromes; inflammatory response; reactive oxygen species; extra virgin olive oil.

## Abstract

Lutein, a plant-derived xanthophyl-carotenoid, is an exceptional antioxidant and anti-inflammatory constituent found in food. High dietary intake of lutein is beneficial against eye disease, improves cardiometabolic health, protects from neurodegenerative diseases, and is beneficial for liver, kidney, and respiratory health. Lutein protects against oxidative and nitrosative stress, both of which play a major role in long COVID and mRNA vaccination injury syndromes. Lutein is an important natural agent for therapeutic use against oxidative and nitrosative stress in chronic illnesses such as cardiovascular and neurodegenerative diseases and cancer. It can also potentially inhibit spike protein-induced inflammation. Rich dietary supplementation of lutein, naturally derived in non-biodegradable Extra Virgin Olive Oil (EVOO), can most optimally be used against oxidative and nitrosative stress during post-COVID and mRNA vaccination injury syndromes. Due to its high oleic acid (OA) content, EVOO supports optimal absorption of dietary lutein. The main molecular pathways by which the SARS-CoV-2 spike protein induces pathology, nuclear factor kappa-light-chain-enhancer activated B cells (NF-κB) and activated protein (AP)-1, can be suppressed by lutein. Synergy with other natural compounds for spike protein detoxification is likely.

## 1. Introduction

Lutein is a fruit- and plant-derived xanthophyl-carotenoid with remarkable antioxidant properties. ^1^ The daily dietary intake of lutein is considered of important medical value as it combats a wide range of pathologies including diseases of the eyes, nervous system, heart, skin, and many others. ^1^^–^^3^ Moreover, an increased concentration of lutein in the blood is positively associated with a) increased respiratory function and b) decreased mortality from respiratory diseases. ^4^ A recent study highlights the urgency of implementing biopharmaceutical-nutrient rich dietary components, including lutein, in order to improve immune function and enhance antiviral activity against SARS-CoV-2 infection. ^5^ Another study focusing on the use of a carotenoid-rich diet against viral infections and disease remarkably reveals the need for lutein consumption as a non-provitamin A nutrient to combat the severe pathologic symptoms of COVID-19 (for full review see Ref. 6). However, although lutein is found abundantly in many consumable plants, including green vegetables, carrots, microalgae, and others, due to its low bioavailability, bio-accessibility and stability, these food sources are not adequate to provide the quantity needed to treat disease. ^7^ One way to overcome the low lutein concentration obtained by diet, and, in order to achieve effective doses against disease, is the use of nutritional supplements containing lutein.

However, the previous clinical studies focusing on the use of lutein supplementation against eye disease do not give clear results. Although high blood concentrations of lutein are achieved in the treated patients with eye disease, there are ambiguous therapeutic effects obtained from these studies. Therefore, the lutein-supplementation approach raises concerns about the efficacy of this nutrient for treating disease. ^8^^,^^9^

Furthermore, lutein as a hydrocarbon carotenoid is transported throughout the body mainly via the low-density lipoproteins (LDL) and high-density lipoproteins (HDL), and to a lesser extent via very low-density lipoproteins (VLDL). ^10^ Therefore, the levels of these lipoproteins in the organism can have either a positive (when in high amounts) or a negative (when in low amounts), impact on lutein’s availability and biodistribution, as shown in human studies on eye health. ^11^^,^^12^ As lutein, due to its hydrophilic nature, is highly distributed in adipose tissue, it seems that obesity can reflect its lower bioavailability and accumulation in the macular pigment. ^13^^,^^14^ Previous human clinical studies have revealed an inverse association between body mass index and carotenoid concentration (including lutein) in serum. ^15^

In this review, we first investigate lutein’s health properties and the mechanisms of its activity. Furthermore, we analyze the antioxidant and anti-inflammatory role of rich food-based lutein intake in order to minimize oxidative stress and oxidant pathways induced by the spike protein in long COVID ^16^ and post mRNA vaccination associated diseases such as myopericarditis. ^17^ Moreover, we investigate the optimum bioavailability of lutein in food combinations that can offer the best effective doses of lutein in order to treat these disorders. Nitrosative stress that accompanies oxidative stress is one of the primary targets of lutein.

Extra virgin olive oil (EVOO), under preferable conditions of purification and storage, can provide a synergistic therapeutic effect and an optimum carrier for a lutein-enriched diet to treat post-COVID and mRNA vaccine injury syndromes. We conclude with a brief review of other naturally derived compounds that can act in synergy with lutein to decrease SARS-CoV-2 spike protein activity and can potentially provide a joint therapeutic effect in post-COVID and mRNA vaccination injuries.

## 2. Long COVID and mRNA vaccination injury syndrome

Long COVID is a unique compilation of diseases due to multi-organ damage occurring in a high percentage of patients who have suffered from COVID-19. ^18^ The pathophysiological mechanisms provoking this set of diseases includes autoimmunity, endothelial damage, gut microbiome irregularities, and increased inflammation that lingers long after SARS CoV-2 infection. On the other hand, post-vaccination syndrome is a recognized compilation of diseases that include similar if not identical pathophysiological mechanisms that occur after COVID-19 (including mRNA) vaccination against SARS-CoV-2. ^19^ However, it should be noted that, despite the intense mRNA vaccination policy over the population, it is the unvaccinated individuals nowadays who have a lower risk of developing long COVID, driving to a reduction of occurrence for this type of disease syndrome in this population. ^20^ In this report, we focus on COVID-19 mRNA vaccination injuries to identify plausible therapeutic benefit of lutein. Long COVID and mRNA vaccination injuries share pathogenic activity of the SARS-CoV-2 spike protein and its engagement with the angiotensin converting enzyme 2 (ACE2) receptor. The engagement of spike protein with the ACE2 receptor is the initiator of disease for both conditions. The SARS CoV-2 spike protein, either originating from the virus or coded within the cells from the synthetic mRNAs, is distributed throughout the immune system via exosomes to manifest as disease symptoms, especially towards causing cardiological diseases, including myocarditis, pericarditis, peri-myocarditis and myocardial infarction. ^21^^–^^23^ The similarities between the pathophysiological mechanisms causing disease in long COVID and mRNA vaccination injuries are described briefly in later sections in relation to the plausible therapeutic activities of lutein.

## 3. The therapeutic properties of lutein’s biologic activity against disease

Amongst the 600 carotenoids that are beneficial for human health and the 20 carotenoids regularly detected in human blood, the tetraterpenoid-pigment, non-provitamin A, xanthophyl-lutein, has exceptional pharmacological activities. These activities are based on the compound’s remarkable antioxidant effects and enhanced properties for scavenging ROS. However, lutein’s abilities to treat disease go beyond the antioxidant and ROS scavenging properties and go far deeper into mechanisms of molecular pathogenesis and inhibition. Comprehensive pharmacological activities of lutein are described in the studies of Kim et al. ^24^ and Algan et al. ^25^ Amongst the most important therapeutic effects, summarized in Table 1, are the anti-cancer effects and protection against cardiac complications and neurodegeneration, in addition to immunomodulation effects and the inhibition of pro-inflammatory responses (produced by interleukin-6 (IL-6), tumor necrosis factor-α (TNF-α) and IL-1β) and chronic inflammation. These therapeutic effects are some of many that can prove useful for the treatment of post-COVID and mRNA vaccination injury syndromes. ^21^^,^^26^

**Table 1.** A selection of specific therapeutic effects and potential alleviation of disease systems resulting from the activity of lutein.

Therapeutic effect of lutein	Mechanisms of lutein activity to alleviate pathogenesis of disease
Anti-cancer	Regulation of apoptosis and angiogenesis. ^27^ Cell cycle arrest, apoptosis of cancer cells, inhibition of cancer cell proliferation. ^28^
Cardiovascular: Cardio-protection Cardio-metabolic diseases (Health benefits provided with a lutein enriched diet)	Lowers inflammation in coronary artery disease associated atherosclerosis. Lowers levels of IL-6 in monocytes and exerts anti-inflammatory activity by lowering IL-1β and TNF-α. ^29^ Lowers stroke incidence and mortality from cardiovascular disease and improves metabolic syndrome by reducing ROS and hyperinsulinemia. ^2^ Prevention of cholesterol build-up, reduction of blood pressure, reduction of arterial thickening, reduction of oxidized LDL. ^30^^,^^31^
Oculo-protection and prevention of eye disease. Lutein-rich diet correlates with diminished risk of age-related macular degeneration-improves eye vision. (Preferably provided with a suitable source in a lutein-enriched diet ^35^)	Inhibition of nuclear cataracts. ^32^ Restoration of retinopathies due to antioxidant activities. ^33^ Filters blue light and reduces photoreceptor cell damage. ^34^
Combats neuro-degenerative diseases. Improves cognitive function. Reduction of Alzheimer's disease (AD) mortality risk in the elderly. ^36^ Protection from severe traumatic brain injury (Lutein rich food).	Reduction of oxidative stress. Inhibition of Nuclear factor kappa-light-chain-enhancer activated B cells (NF-κB) signalling pathway and activation of Nrf2. ^37^ Anti-oxidation exerted via the nuclear factor erythroid 2–related factor 2 (Nrf2) and ICAM-1 downregulation. Downregulation of cyclooxygenase-2 activity. ^38^
Stabilization of high glucose level effects in immune cells.	Reduction of oxidative stress induced by glucose. Reduction of nuclear factor-kappa beta (NF-κB) activity. ^39^
Inhibition of obesity	Inhibition of adipocyte differentiation. Delay of adipose cells at G0/G1 phase of cell cycle. ^40^
Diabetes: Diabetic nephropathy. Diabetic retinopathies.	ROS scavenging. Reduction of serum and urine urea and creatinine. Decrease of TNF-α, IL-6 and IL-1 in renal tissues. Restoration of pro-inflammatory cytokines to normal in renal tissues and restoration of oxidative/nitrosative stress biomarkers. ^41^ Improvement in visual acuity, contrast sensitivity and macular oedema in diabetic retinopathy patients due to protection from visible light. ^42^
Immunomodulation of inflammation.	Reduction of proinflammatory cytokine levels. Inhibition of chronic inflammation (increase of Il-10). ^43^ Inhibition of hyperosmocity-induced secretion of IL-6 through the deactivation of p38, JNK and NF-κB pathways. ^44^
Protection of liver health: Prevention of non-alcoholic steatohepatitis (NASH) evolution from non-alcoholic fatty liver disease (NAFLD).	Decrease of insulin resistance and lipogenesis. Prevents triglyceride synthesis, free fatty acid and cholesterol deposition and lipid peroxidation. Reduction of cytokine inflammatory response, and oxidative stress. ^45^ Decrease of hepatic TNF-α and NF-κB DNA binding activity in *in vivo* studies. ^46^
Heart and kidney axis: Prevention of cardiac and renal injuries.	Improves glucose tolerance. Restores balance of polyol pathway. Decreases malondialdehyde levels and increases reduced glutathione levels in the serum, heart and kidney. Modifies the antioxidant enzymatic activities of catalase, glutathione peroxidase, reductase and transferase, and superoxide dismutase in diabetes. ^47^
Anti-viral effects.	Inhibition of hepatitis B virus (HBV) transcription *in vitro.* ^48^ Inhibition by binding to Nipah virus protein *in silico.* ^49^ Inhibition of vaccinia virus *in vitro.* ^50^
SARS-CoV-2 spike protein potential neutralization properties. Obtained from molecular docking and molecular dynamics in *in silico* simulation studies.	Direct binding to the Lys417Asn position of spike protein, part of the spike-human angiotensin converting enzyme 2 (ACE2) interface (Wuhan lineage variants) *in silico.* ^51^ Highest binding affinity amongst polyphenolic compounds (including quercetin and luteolin) against spike protein. Potential inhibition of SARS-CoV-2 protease active sites. ^52^ FDA selected drug candidate to block ACE2 binding affinity of spike protein. ^53^
Potential anti-COVID-19 activity.	Reduction of oxidative stress and inflammatory injury. Lowering of pro-inflammatory cytokine mediators including IL-16 and TNF-α receptor-1. ^6^

Summarizing the literature evidence of lutein’s therapeutic effects presented in Table 1, the lipophilic, highly antioxidant molecule lutein exerts its activity on many organs of the body, including the brain, the heart, the kidneys, and the liver. Due to its structural properties (lutein is a dihydroxy carotenoid), the hydroxyl groups make lutein more hydrophilic than other xanthophylls (e.g., zeaxanthin) and increase its polarity in comparison to other carotenoids. It is therefore described in the literature that, due to the increased hydrophilicity and polarity, lutein can act more effectively than other carotenoids as a scavenger and neutralizer of ROS to more adequately prevent overwhelming damage in tissues. ^54^

## 4. Beyond the ROS scavenging properties of lutein. Relations to alleviations of SARS-CoV-2 spike protein-induced pathology

Apart from the enhanced scavenging properties of lutein, further studies show that this molecule exerts its anti-inflammatory activity by intervention in Toll-like receptor (TLR) signaling. Specifically, the Lin et al. study on broilers ( *in vivo* and *in vitro* experiments) reveals that lutein exerts anti-inflammatory activity by reducing activity of the TRL4/myeloid-differentiation-factor 88 (MyD88) signaling pathway. ^55^ The *in vitro* downregulation of TLR4/MyD88, combined with the reduction of *TLR4* gene expression shown *in vivo*, explains why lutein produces an anti-inflammatory effect. This is by the reduction in expression and secretion of IL-1β and IL-6, and the increased expression and secretion of the anti-inflammatory cytokines IL-10 and Il-4. This study and several other studies also show downregulation of NF-κB signalling by lutein. ^44^^,^^48^^,^^55^^,^^56^ Relevant to the pathogenesis caused by the SARS-CoV-2 spike protein, the upregulation of TLR4/MyD88 signalling is probably one of the key factors that induce excess inflammation through upregulation of IL-6, IL-1β and NF-κΒ. ^57^^,^^58^ Moreover, the key phosphorylating pathways that are intensely activated through TLRs by the SARS-CoV-2 spike protein and are said to contribute substantially to the protease’s pathogenesis are p38 mitogen activated protein kinase (MAPK) and the c-Jun NH2-terminal kinase (JNK). ^59^ p38 MAPK becomes activated by both TRL4 and TLR2 pathways. ^60^

We have recently published a proposed approach to assist the human body in catabolism of the spike protein using three over-the-counter oral medications/supplements: nattokinase 2000 FU (100 mg) twice daily, bromelain 500 mg a day, and curcumin 500 mg twice a day. It is conceivable that serratiopeptidase and alkaline serine peptidase could also have a proteolytic effect on the spike protein. This base of detoxification therapy is proposed for 3-12 months or more while research continues and additional drugs are used clinically as indicated in each case. Addition of dietary lutein is very reasonable given the current state of knowledge in the field. ^61^^,^^62^

Spike protein, as well as its fragments released with detoxification, promote inflammation by stimulating the TLR2 and the TLR4 signaling pathways and hence the subsequent NF-κB response. ^57^^,^^63^ End-stream of TLR4 and TLR2/p38 MAPK-dependent activation is the activation of the cAMP response element—binding protein (CREB). ^64^ CREB in turn, when activated by p38 MAPK, regulates the gene expression of activated protein (AP)-1. ^65^ AP-1 is a family of proteins and consists of transcription factor clusters implicated in inflammatory diseases, ^66^ the development of neurodegenerative diseases, ^67^ and in the development of cancer. ^68^ Recently, our team has shown that SARS-CoV-2 spike protein, by stimulating the p38 MAPK dependent pathways, promotes the transcription of AP-1. ^59^ The over-regulation of AP-1, however, can be lessened by lutein, according to *in vitro* studies.

Oh et al. ^69^ investigated the role of lutein in suppressing the p38 MAPK and JNK pathways and concluded that lutein suppresses AP-1 activation and inflammatory processes through these pathways. The authors used LPS-induced macrophage and keratinocyte cellular systems to show that lutein suppressed the generation of IL-6 and production of cyclooxygenase 2 (COX2), a potent inflammatory and cancer-inducing enzyme, via both the interferon-γ (IFN-γ) and TNF-α pathways. ^70^ In these experiments, lutein also suppressed the rising levels of matrix-metalloproteinase-9 (MMP-9). MMP-9 is a tissue remodelling protease, and increased activity is implicated in the progression of inflammatory disease (arthritis and diabetes), cardiovascular diseases (fibrosis, hypertension, and myocardial infarction), and cancer. ^71^ In this regard, the activation of AP-1 and NF-κB constitute important factors regulating the transcription and translation of MMP-9.

In summary, lutein acts as a strong antioxidant – ROS scavenger agent and suppresses the p38/JNK-dependent activity of AP-1 transcription factors and pro-inflammatory cytokines, including MMP-9 and is well positioned as an adjunct dietary strategy to assist in base spike protein detoxification.

**Figure 1.**
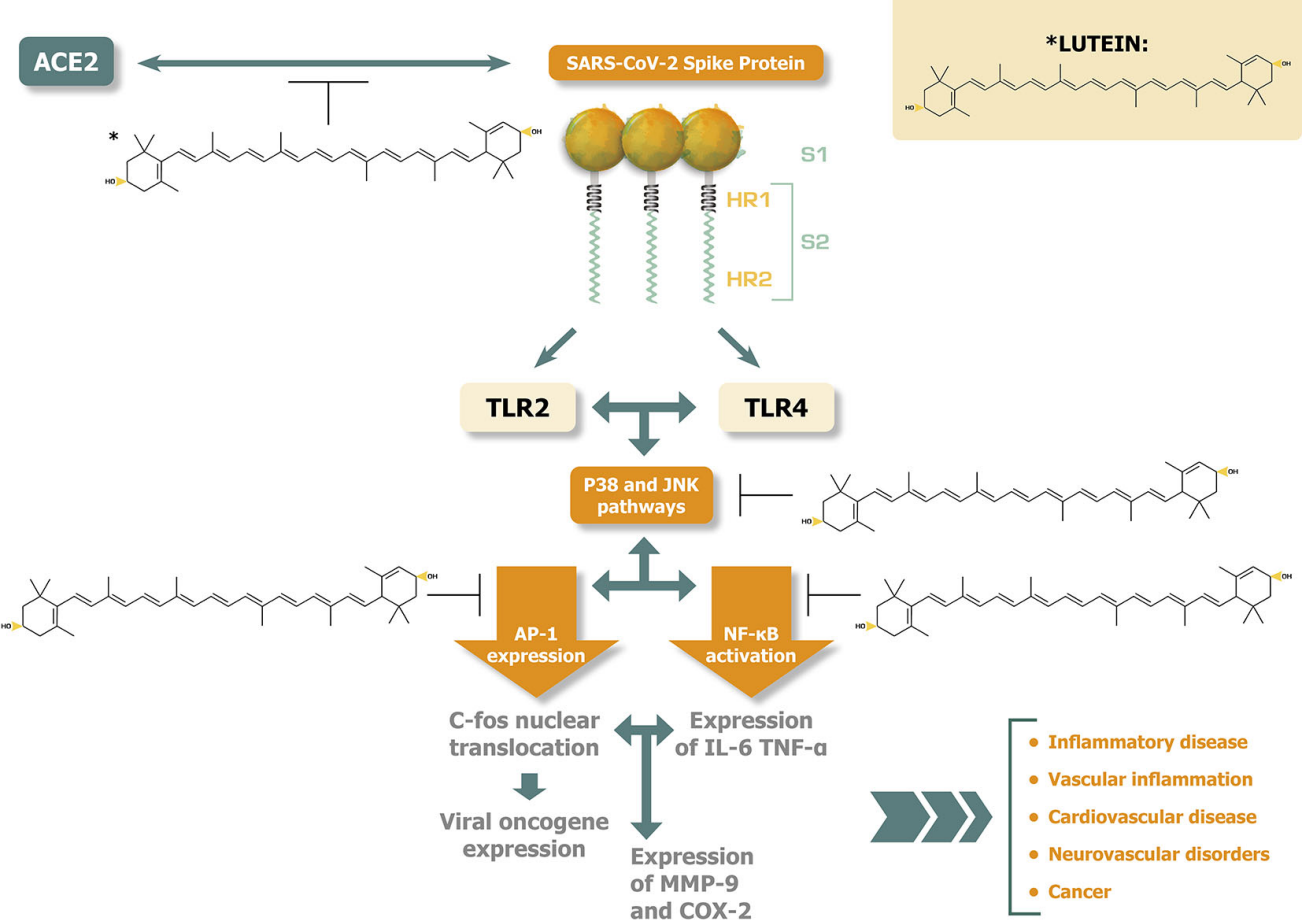
Lutein inhibits at several points p38 MAPK and JNK activations, potentially alleviating disease consequences caused by SARS-COV-2 spike protein. ^59^^,^^69^^–^^74^

Figure 1 illustrates the potential disease alleviating effects of lutein against the activation of p38 MAPK and JNK pathways and the related pathogenic responses induced by SARS-CoV-2 spike protein interactions.

## 5. Lutein, oxidative stress, long COVID syndrome, and mRNA vaccination injuries

Long COVID syndrome is a group of diseases occurring when the pathogenesis of SARS-CoV-2 and spike protein are manifested for a prolonged time after the resolution of infection. ^75^ Apart from the regular symptomatology of COVID-19, manifested also in long COVID syndrome (fatigue, headache, myalgia, etc.), the neurologic, ^76^ psychiatric ^16^ and cardiological ^77^^,^^78^ complications of long COVID syndrome, amongst many others (renal, respiratory, skeletal muscle, etc.), ^78^ can be severe, requiring repurposing of drugs. One of the main mechanisms causing the disease manifestations in long COVID syndrome is oxidative stress resulting in tissue damage, and the lowering of antioxidant defenses. ^16^

The mRNA vaccines against COVID-19, similarly to long COVID, can result in over-production of inflammatory cytokines that finally produce a multi-organ hyper-inflammatory response involving the cardiovascular system, the brain and the liver, as shown in human studies, ^79^^–^^82^ amongst other pathologic findings found from *in vivo* and *in vitro* studies. ^83^ Remarkable is the emergence of autoimmune and immune-mediated conditions after the mRNA vaccinations. ^17^^,^^82^ Regarding the cardiological complications, Giannota et al. analyzed the role of oxidative stress in relation to the angiotensin/aldosterone hormonal axis which becomes upregulated by the circulating mRNA-expressed spike protein. ^84^ Cases of pericarditis, associated with increased oxidative stress, can be attributed to the SARS-CoV-2 spike protein expressed by mRNA vaccination. ^17^ In relation to oxidative stress, Table 2 provides a collection of representative studies, found by searching thoroughly in PubMed and ScienceDirect databases, that relate to long COVID and mRNA injury syndromes. These studies predominantly highlight that both syndromes are related to the elevation of oxidative stress and thus to the weakening of antioxidant defenses. The findings of increased inflammatory responses are included in Table 2, as these are considered to be the result of excessive oxidative stress in the organism.

**Table 2.** A collection of representative studies highlighting the role of oxidative stress and subsequent tissue damage in the long COVID and mRNA vaccination injury syndromes.

Scope of study	Findings on oxidative stress and inflammatory response
**Studies on long COVID in relation to oxidative stress**	
Analysis of oxidative stress biomarkers in long COVID patients after a previous mild SARS-CoV-2 infection.	Malondialdehyde serum levels (MDA) (lipid peroxidation product) remain high in long COVID patients compared to healthy controls. ^85^
Inflammatory protein profiling in long COVID patients, 5 months to 1 year after COVID-19 hospital discharge.	Systemic inflammation was evident in long COVID patients with upregulated IL-6 and neuroinflammation. C-terminal fragment of agrin protein found elevated. Breakdown of agrin indicates cardiomyocyte and neuromuscular junction damages. ^86^ Prediction of neuropsychiatric symptoms correlated with antioxidant/pro-oxidant imbalance. ^87^
Investigation of myalgic encephalomyelitis/chronic fatigue syndrome in long COVID syndrome.	Redox imbalance. Increased levels of peroxides and superoxide. Elevated levels of pro-oxidants including nitrogen species. Nitrosative stress and abnormally high nitric oxide (NO) levels. High levels of homocysteine indicating impairment of reverse trans-sulfation enzyme activities. Inability to generate adenosine triphosphate. ^88^
Investigation of long COVID chronic fatigue, somatic and mental symptoms.	Increased peak body temperature predicting high C-reactive protein (CRP) levels. Low glutathione peroxidase (GPX) and zinc levels. Increased myeloperoxidase (MPO), NO and lipid peroxidase-associated aldehyde levels. Neuro-immune and neuro-oxidative stress associated inflammation. ^16^
Investigations of cognitive impairment in long COVID and associations with inflammatory markers	Cognitive impairment-associated neuro-oxidative stress inflammation parallels increased cytokine profile including IFN-α, TGF-β, TNF-β, IL-6, IL-7, IL-13, IL-15 and G-CSF. Cognitive impairment is significantly associated with CRP and D-dimer levels. ^16^^,^^89^
**Studies on mRNA vaccine injury syndrome in relation to oxidative stress**	
Evaluations on mRNA-induced acute-pericarditis and myopericarditis in young adults and associations with oxidative stress.	Myopericarditis was associated with increased levels of troponin I, D-dimers and high sensitivity CRP (hsCRP). Oxidative stress index (TOS/TAS ^*^), ^90^^,^^91^ and NO levels were lower in myopericarditis group compared to acute-pericarditis and control groups, indicating an inflammatory and pro-coagulant condition in the myopericarditis. ^17^
Investigation of the mRNA vaccination effects of oxidative stress and DNA damage on blood mononuclear cells (BMC) in the young (aged 27-44 years) and the elderly (aged 80-88 years) population groups.	Increased oxidative and DNA damage in all population groups. Accumulation of double strand breaks (DSB) in the elderly peripheral BMCs as compared to younger population occurring with every mRNA (first and second shot) vaccination. Pro-oxidant/antioxidant imbalance was associated with reduced humoral response after mRNA vaccination. This was linked with immune senescence in the elderly population. ^92^^,^^93^
Investigations on arrythmia and myocarditis.	Autopsies indicate acute arrythmogenic cardiac failure/lethal complication of mRNA vaccination. ^79^^,^^80^^,^^94^^,^^95^ Possible redox/pro-oxidant/antioxidant imbalance resulting in sudden death. ^96^
Investigations on electrocardiogram abnormalities after the mRNA vaccinations	Myopericarditis association with IL-18 NLRP3 inflammasome activation. ^95^ Potential upregulation of IL-18/activator protein (AP-1) redox sensitive signaling via p38 MAPK activation. ^97^ Possible pro-oxidant stimuli, leading to vascular damage. ^94^ Mass (4928 students) screening study on electrocardiogram (ECG) parameters ^98^ revealed that depolarization and repolarization of heart (QRS duration and QT interval) reduced significantly after the second dose of mRNA vaccine, whilst the heart rate was increased. Arrythmia cases can be associated with Na ^+^/Ca ^2+^ exchanger redox imbalance, sarcolemma depolarization defects and ventricular arrythmia. ^96^
mRNA vaccination related to autoimmune disorders that result in hair loss	Patients in complete remission (mean 1.8 years) from alopecia areata (AA), [(Severity of Alopecia Tool (SALT): 0 (S0)], deteriorated after the first mRNA vaccine, showing stability of AA symptoms after the subsequent doses. One patient showed a booster effect of AA symptoms after subsequent doses. ^99^ Possible oxidative stress triggering, resulting in inflammatory process activation and hair loss. ^100^

* TOS, total oxidant status (mmol H2O2 Eq/L), TAS, total antioxidant status (mmol Trolox Eq/L).

Summarizing the literature collection of studies presented in Table 2, both long COVID and mRNA injury syndromes present with pathologic outcomes that are related to pro-oxidant/antioxidant (redox) imbalance. ^94^ The redox homeostatic imbalance lowers the antioxidant defenses and causes excessive tissue damage. This can be lethal when the heart, the vasculature and the brain are affected. ^80^^,^^96^ Oxidative stress influences the activation of the redox-sensitive pro-inflammatory pathways of NF-κΒ and AP-1, and the activity of their related transcription factors portends the outcome of the related pathologies. This is shown on a molecular and clinical level from previous studies on atherosclerosis. ^94^^,^^101^^,^^102^ How the development of oxidative stress and the subsequent production of ROS detrimentally influence the development of redox imbalance and thus result in an arrhythmogenic heart is described in detail by the Dashwood. ^96^ However, beyond ROS, there is also the issue of reactive nitrogen species (RNS) under conditions of oxidative stress. ^96^ The activity of molecules involved in maintaining ROS and RNS homeostasis become imbalanced during oxidative stress conditions.

## 6. The ROS and RNS imbalance during long COVID and mRNA injury syndromes – The potential therapeutic role of lutein

The studies that have investigated the levels of NO a) in the serum of patients suffering from myopericarditis as a consequence of mRNA vaccination against SARS-CoV-2, ^87^ and b) in the blood of patients suffering from myalgic encephalomyelitis and chronic fatigue syndrome as often encountered in long COVID ^88^^,^^103^ find that they deviate from normal values. The abnormal values of NO indicate a ROS and RNS imbalance producing oxidative and nitrosative stress. Therefore, the following subsections are dedicated to analysing the potential implications of ROS and RNS imbalance with respect to post-COVID and mRNA injury pathological developments. Moreover, lutein can potentially find its place in therapy as a diet-provided nutrient to normalise the pathologic levels of NO in post-COVID syndrome and mRNA vaccination induced injuries.

### 6.1 The redox buffering mechanism that protects from ROS-induced arrythmias

The mechanisms of ROS and RNS production involve a plethora of molecules and an interacting enzyme cascade. The ROS are predominantly produced by oxidase enzymes such as xanthine oxidase (XO) and nicotinamide adenine dinucleotide phosphate (NADPH) oxidases (NOX) during mitochondrial oxidative metabolism. ^104^^,^^105^ Moreover, superoxide dismutase (SOD) consumes superoxide anions (O _2_^-^) leaked from the electron chain to form hydrogen peroxide (H _2_O _2_). Peroxides in turn react with transition metals to produce hydroxyl radicals (OH ^.^).

Furthermore, XO produces electrons from purine degradation that contribute to H _2_O _2_ synthesis. NOX may also produce superoxide anions; however, both NOX and XO produce low levels of ROS in cardiac cells. ^96^ RNS are produced by nitric oxide synthase (NOS). NOS, apart from nitric oxide (NO), also produces superoxide (O _2_^-^). NO and O _2_^-^ can react with each other to produce the highly reactive product, peroxynitrite (ONOO ^-^). ^105^

Surprisingly, low dose administration of peroxynitrite can act as a buffer molecule to prevent ventricular arrythmia. Experiments in animals have shown that intracoronary infusion of peroxynitrite suppressed ischemia and reperfusion (I/R)-induced ventricular arrhythmias. ^106^ However, I/R induced ventricular arrhythmia suppression by peroxynitrite infusion was accompanied by an increase in nitrate/nitrite (NOx) metabolite levels and a decrease in endogenous superoxide production. Overall, these findings led to the conclusion that, due to the reduced superoxide production after the peroxynitrite infusion, there was adequate NO availability during the induced ischemia experiments that resulted in protection from ventricular arrythmia.

### 6.2 Abnormal levels of NO may contribute to cardiovascular and brain pathologic conditions in post-COVID and mRNA injury syndrome

The normal production of NO by neuronal NOS (nNOS) and endothelial NOS (eNOS) is beneficial and leads to homeostasis in the neural system, including the brain ^107^ and the cardiovascular system. ^108^ An abnormal rise of NO in the organism, however, predominantly points to cytokine-mediated excessive release of NO by macrophages, and this is related to inflammatory disorders and excessive neurotransmission. ^109^ Remarkable is the evidence of the pathogenic relationships between elevated levels of NO and autoimmune conditions such as rheumatoid arthritis ^110^ and cognitive impairment and dementia. ^107^ An excess of NO in the brain during oxidative stress, and hence excess of oxygen species generation, gives more chances for NO to produce peroxynitrite, which is highly linked to the pathogenic predisposition to Alzheimer’s disease (AD). ^111^ Furthermore, the increased levels of NO create more chances for stroke episodes under conditions of extensive RNS-induced mitochondrial enzyme damage. ^112^

On the other hand, unusually low concentrations of NO in the organism are a strong indicator for endothelium dysregulation and damage, for predisposition to severe cardiovascular disease, and other cardiovascular complications, if these are not already pathologically prevailing. ^108^ During oxidative stress, low levels of NO predict that most of the NO synthesized by either eNOS or nNOS, or by inducible NOS (iNOS) (a special NOS induced under immune stimulation), is converted to peroxynitrite.

The catastrophic effects of the reduced levels of NO for the cardiovascular system are well known and include vasoconstriction, platelet aggregation, vascular endothelium damage, and enhancement of adhesion molecule (ICAM-1, VCAM, etc.) activity. These are fully documented by Raddino and colleagues. ^108^ Furthermore, peroxynitrite accumulation leads to a further increase of ROS. An excess of peroxynitrite and ROS leads to the formation of S-nitrosoglutathione (GNSO) and oxidized glutathione (GSSG). The oxygen radicals and GSSG have a direct effect on cardiac ryanodine receptor type 2 (RyR2) activity to produce an arrhythmogenic phenotype that can eventually result in heart failure. The full description of how a ROS/RNS imbalance causes an arrhythmogenic phenotype is described in the study of Dashwood and coworkers. ^96^ Briefly, the ROS/RNS imbalance and the reactivity of nitrogen and oxygen species produced out of this imbalance (accumulation of peroxynitrite, GSSG, H _2_O _2,_ OH ^.^), through their interaction with the RyR2 receptor found on the membranes of sarcoplasmic reticulum (SR), result in direct modifications of the protein’s tertiary structure by inducing disulfide bond formations, e.g., S-nitrosylations and S-glutathionylations on the RyR2 residues. The deformations on the RyR2 tertiary structure caused by the ROS/RNS imbalance are coupled with indirect effects on its structure, secondary to oxidation of Ca ^2+^calmodulin-dependent protein kinase II (CaMKII) and protein kinase A (PKA). The activation of these enzymes ultimately results in the phosphorylation of RyR2 residues, the dissociation of the regulatory CaM ligand from RyR2, and to other alterations of the RyR2 protein structure. These structural changes culminate in the deregulation of intracellular Ca ^2+^ gating in SR, otherwise controlled by RyR2 structural intactness. Due to these structural changes, the open probability ( *P _o_*) of RyR2, which reflects the maintenance of the controlled release of Ca ^2+^ in SR, becomes altered and increases. The overall physiologic effect is Ca ^2+^ diastolic leak in SR, which leads to arrhythmogenic heart failure.

The arrhythmogenic cardiomyopathies (AC) are a group of heart diseases that can be the result of either genetic or acquired causations. Some of the main acquired causative factors that produce the AC phenotype are infectious myocarditis and toxicity-related cardiomyopathy. The most severe consequences of AC are ventricular ectopy, tachycardia, and unexplained sudden death. ^113^ In the study of Schwab and coworkers, ^80^ the cause of death after mRNA vaccination was attributed to acute arrhythmogenic cardiac failure that resulted from myocarditis. Although in this study, specific markers that evaluate the role of oxidative stress in relation to disease and sudden death were not included, reviewing the study of Dursun and colleagues ^17^ can be enlightening in this respect. This study concludes that the levels of nitric oxide and oxidative stress index (OSI) are lower in the mRNA-related myopericarditis cases as compared with the acute-pericarditis cases. The OSI system employed by researchers in this study is a tool for the evaluation of the total oxidant status (TOS) over the total antioxidant status (TAS) in the organism. ^90^^,^^91^ The assessment of OSI is based on the oxidative stress produced during acute ischemia/reperfusion (I/R) injury of the vasculature in animals. During the I/R-induced vascular tissue injury, the total concentrations of oxidant molecules (TOS), measured in mmol H _2_O _2_ Eq/Lt, become higher than normal. However, during vascular injury, the total concentration of antioxidant molecules (TAS), measured in mmol Eq/Lt, is much more enhanced than the elevation of TOS. This implies an overall drop of the OSI, a marker which is significant for the estimation of vascular tissue damage due to oxidative stress. ^90^^,^^91^

It is thus possible that, in fatal myocarditis cases caused by mRNA vaccination, ^80^ the arrhythmogenic cardiac failure was the result of excessive oxidative stress and therefore a ROS/RNS imbalance, as described by the research groups of Dashwood et al. ^96^ and Protonotarios and colleagues. ^113^ Moreover, the SARS-CoV-2 spike protein in its whole structure (trimer), is detected in the serum of patients developing myocarditis (accompanied with elevated levels of troponin) after mRNA vaccination, and not in the symptom-free vaccinated control cases. ^114^ It can thus be assumed, again, that the toxicity inducing cardiomyopathy and causing the pro-oxidant/antioxidant imbalance in the organism, ultimately resulting in AC and death, ^80^ is the spike protein expressed from mRNA vaccination. This conclusion is strongly supported by the study by Protonotarios A & colleagues. ^113^

In this regard, the circulating levels of NO in the serum of the mRNA-injured patients are measured in low concentrations. ^17^ However, the levels of NO measured in the serum of post-COVID patients suffering from myalgic encephalomyelitis and chronic fatigue syndrome are found in abnormally high concentrations, indicating nitrosative stress. ^88^ Nevertheless, more studies are needed to look specifically at the role of nitrosative stress in these conditions in the long COVID scenario.

The elevated levels of NO in the population of long COVID patients can also be a contributing factor for cardiovascular disease. When the NO concentration is abnormally high in the blood and persists over time, especially in males, there is a higher risk of mortality from cardiovascular disorders. ^115^ On a molecular level, during nitrosative stress, the NO conjugation with oxygen radicals and the subsequent process of peroxynitrite formation leads to the pathogenic nitrosylation of several compounds, including tyrosine residues in proteins to produce 3-nitrotyrosine (3-NT), and also nitrosylation of lipids and DNA.

Accumulation of 3-NT eventually results in cellular death. The molecular relations of the processes of apoptosis, autophagy, pyroptosis, ferroptosis and the newly discovered process of parthanatos, with nitrosative stress and cell death due to 3-NT activity, are described in the study of Wang and colleagues. ^116^ Eventually, the death of cells from nitrosative stress leads to a host of pathologic events, including myocardial hypertrophy and fibrosis, that lead to ischemia and heart failure. Furthermore, inflammation caused by NO, ^109^ and the vascular damage to tissues other than the heart, lead to increased risk of neurological deficits of brain functions. ^107^ It can thus be hypothesized, based upon the available evidence, that both conditions of abnormal NO levels in long COVID (high NO) and mRNA vaccination injuries (low NO) are related to an overall ROS/RNS imbalance in the patients.

Especially vulnerable to SARS-CoV-2 spike protein are the human microglial cells. Clough et al. have shown that the spike protein, apart from producing extensive oxidative stress and an increase in hypoxia inducing factor (HIF1α) expression, also induces a 57% elevation in the expression of NOS in microglia. The authors note that nitrosative stress is one of the major contributors of spike protein-associated neuroinflammation and development of Neuro-COVID. ^117^

## 7. The nutritional supplementation of lutein can potentially alleviate the pathologic levels of NO found in post-COVID and mRNA injury syndromes

Newborn infants provide possibly one of the best models to investigate the effects of a therapeutic agent on oxidative stress. Infants lack adult antioxidant defense mechanisms and therefore suffer from an excess of oxidative reactions due to an enhanced metabolic rate. Furthermore, human cells are unable to synthesize lutein. All these conditions have led investigators to measure precisely the effects of lutein on the alleviation of oxidative stress in infants. Studies on newborn infants have shown that the reduction of oxidative stress and balance of cellular redox potential can be achieved by lutein supplementation. ^118^^,^^119^

Other studies show that newborns also suffer from nitrosative stress complications due to oxidative stress reactions. ^120^ It is therefore reasonable to assume that lutein can provide additional therapeutic benefits in infants due to the complementary balance of nitrosative stress and RNS that will result from the balance of oxidative stress produced by ROS. ^118^^,^^119^ Furthermore, a specific study investigating the effects of ischemia/reperfusion-induced injury of retinal tissue indicates that the neuroprotective role of lutein results from balancing the RNS. The study shows clearly that lutein achieves an alleviation of oxidative damage and nitrosative stress concurrently. The study used the levels of nitrotyrosine as a marker to estimate the relief of nitrosative stress. ^121^

Having the aforementioned studies in mind, the close relationship between oxidative stress and abnormal NO levels, shown in studies included in Table 2, which correlate NO with post-COVID encephalomyelitis ^16^ and mRNA injury-related myopericarditis, ^17^ indicate that lutein can be of therapeutic benefit for both conditions. Lutein can therefore be used as a regulatory molecule to equilibrate excess oxidative and nitrosative stress locally in harmed tissues and thereby normalize the NO levels in these conditions. This is likely specific to lutein because, in comparison to its structurally close homolog zeaxanthin and other xanthophyll carotenoids, lutein has a unique molecular configuration to achieve free radical scavenging with highest efficiency. ^122^ The free radical species can also be RNS, and, as relevant studies show, lutein has the capacity to reduce by 50% the generation of NO produced during inflammation, while at the same time reducing mRNA expression and translation of iNOS by 75%. ^37^^,^^123^ In addition to its beneficial impact on the redox status of NO, lutein also promotes expression of antioxidant enzymes such as heme-oxygenase-1 and NAD(P) H dehydrogenase (quinone-1).

As already discussed, lutein’s anti-inflammatory activity is mainly centralized on the reduction of NF-κB activity via the inhibition of p38 MAPK, JNK and Akt pathways. Specifically, for neural tissues, this has been convincingly shown in the Wu W & colleagues’ study. ^37^ The researchers have used microglial cell lines to demonstrate that the anti-inflammatory and neuroprotective activity of lutein is exerted through the activation of the extracellular signal-regulated kinase 1/2 (ERK1/2) mediated NF-E2-related factor 2 (Nrf2) pathway. On the other hand, the neurotoxicity of SARS-CoV-2 spike protein is mainly exerted through the activation of p38 MAPK and JNK pathways that promote NF-κB activation and result in neurodegeneration. ^59^

Therefore, lutein can plausibly serve as a buffer molecule against excess oxidation and nitrosative stress and thereby potentially reduce or even eliminate the type of post-COVID and mRNA injury cases shown in Table 2. This effect may extend from attenuation of oxidative stress and in parallel restoration of levels of NO and reduction of other related toxic compounds produced during nitrosative stress.

Neurotoxicity is induced by the spike protein. ^62^ It is noteworthy here that lutein can buffer the potency and lower the p38 MAPK-related pathways that activate NF-kB, and thus prevent or even provide therapeutic effects on the conditions of dementia and neurodegeneration observed in post-COVID syndrome. ^16^^,^^92^

**Figure 2.**
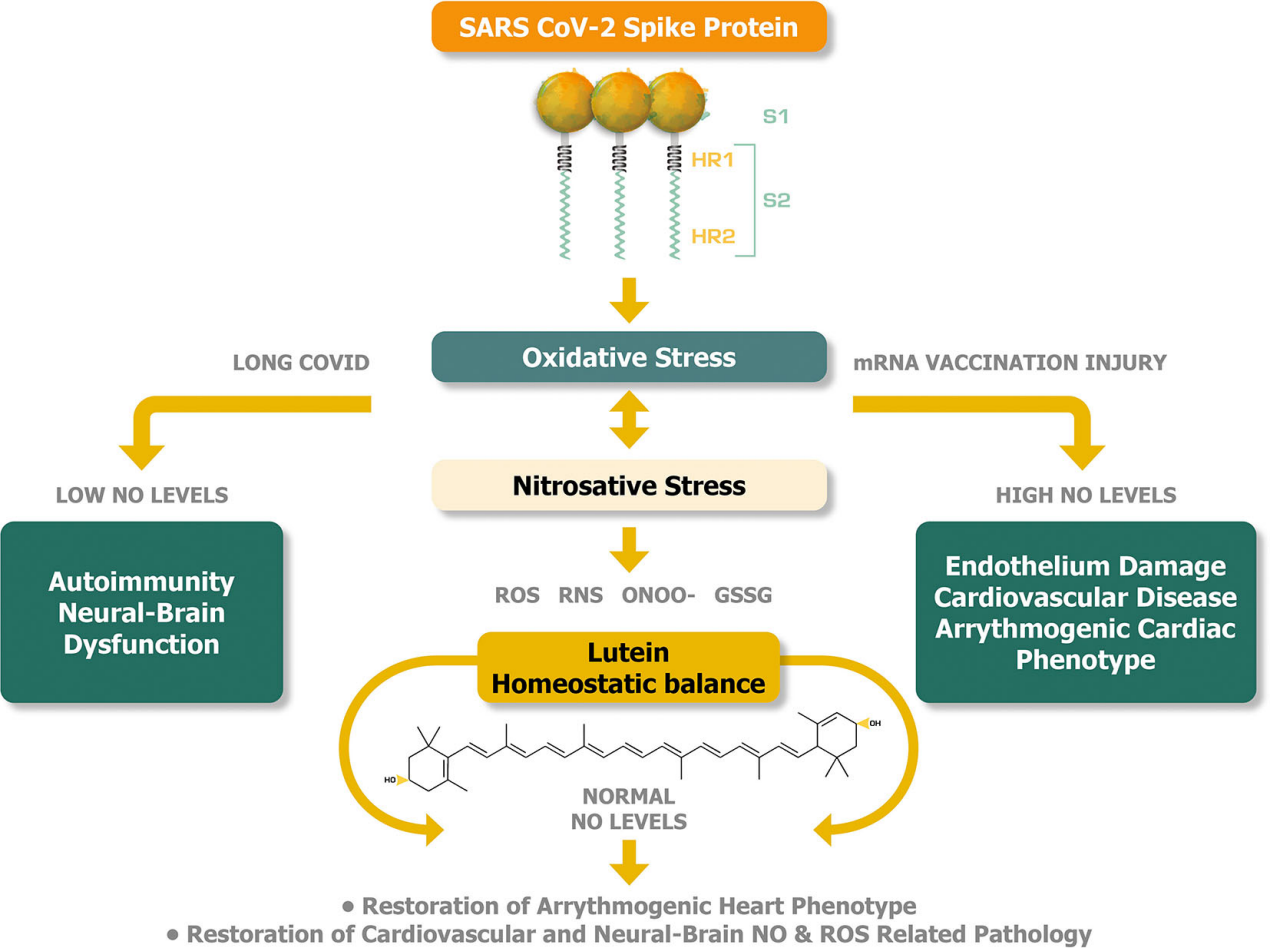
Lutein can potentially offer a homeostatic balance between oxidative and nitrosative stress to alleviate the pathogenesis of long COVID and mRNA injury syndromes. ^16^^,^^17^^,^^37^^,^^80^^,^^88^^,^^96^^,^^108^^–^^123^

Figure 2 illustrates the plausible buffering and therefore potentially therapeutic activities of lutein that can be achieved by the equilibration of abnormal levels of NO in long COVID syndrome and mRNA injury syndromes.

Furthermore, the therapeutic effects of lutein on neurodegenerative disorders may go beyond the compound’s high antioxidant/anti-inflammatory activities. Recent experimental evidence demonstrates that lutein enhances the transfer of heme in microglial cells without causing iron accumulation and deposition. Normal heme and thus iron transport is beneficial for brain functions. It seems that lutein achieves this goal by inducing the expression of mitochondria iron transporter genes. As the authors of the relevant study conclude, the buffering effect of iron transport conferred by lutein may prove beneficial to counter the pathologic excessive iron deposition encountered in neurodegenerative disorders of AD, PD and sideroblastic anaemia. ^124^ Finally, the downregulation of IL-6-dependent pro-inflammatory signalling by lutein may prove beneficial for the treatment of autoimmune disorders of the skin such as alopecia that occurs after mRNA vaccination, ^99^^,^^100^ as well as other IL-6 related autoimmune and inflammatory disorders that may arise due to inflammatory cytokine upregulation. ^44^^,^^99^^,^^100^^,^^125^

## 8. Why lutein-rich food may be ideal

There is convincing evidence to suggest that a lutein-rich diet can be a valuable therapeutic intervention, either preventing onset of disease or alleviating disease symptoms. Amongst the carotenoids, the xanthophylls, and especially lutein (as mentioned previously) has unique structural properties that enable the molecule to perform enhanced scavenging of ROS and RNS (NO, peroxynitrite and N _2_O _3_). ^122^^,^^126^ Moreover, during the inhibitory reactions with ROS and RNS, the energy obtained in lutein’s structure transforms the molecule to its triplet state. However, the lutein molecule has the capacity to release the energy gained as heat and return to its original ground state. In this regard, the regenerative capability of lutein makes it reusable for additional rounds of free radical species elimination, and, thus, its quantity and protective activity can be maintained over time in the tissues. ^127^ Furthermore, the content in lutein of the retinal pigment, also called macular pigment (MP), is derived from dietary consumption. ^11^ Relevant animal studies on primates reveal that the lutein content within MP correlates with the content found in the tissues of the brain, ^128^ and therefore this may be the result of the accumulation of lutein from the diet in the organs. A relevant study that has investigated the content of lutein in the eyes of animals as a result of dietary supplementation corelates the antioxidant and anti-inflammatory effects of lutein against liver injury with the concentrations of lutein measured in the plasma and ophthalmic tissues. ^30^ Moreover, increased plasma concentrations of lutein achieved by increased dietary intake are protective against primary development of atherosclerosis. ^129^ Finally, when lutein is supplemented by intraperitoneal injections in animals and therefore travels by plasma to the renal tissues, it prevents kidney damage caused by ischemia/reperfusion injury. ^130^

On average, as human studies on eye-related disease show, at least 6 mg/day of lutein should be consumed from food to achieve preventative or therapeutic results. ^131^ Unfortunately, lutein has low bioavailability and bio-accessibility from food sources, and its stability depends on the food matrix and presence of other dietary components. The food processing (heat, pH etc) that degrades the structural stability of lutein makes its bioavailability even worse. Therefore, technical advances on microencapsulation are being developed to solve these problems. ^132^ However, clinical studies on age-related macular degeneration (AMD) recommend that the consumption of food-based lutein is the best option, as the administration of supplements for AMD have given contradictory results. ^133^ This may be because high-dose administration of a single xanthophyl-carotenoid like lutein has the effect of altering the plasma and other tissue concentrations of other xanthophylls such as zeaxanthin, and carotenoids such as β-carotene, which are also beneficial for the prevention of human disease. ^134^^,^^135^ Given that the food-based consumption of lutein in western countries such as the USA is remarkably low (1.5 mg/day of lutein), ^136^ other ways of improving human consumption of lutein should be investigated.

Regarding the food matrix and other dietary components that affect its stability, ^132^ the Lakshminarayana and colleagues animal study has found that olive oil (OO) is the optimum “carrier source” to achieve the best postprandial plasma concentrations of lutein that can reach the eyes. ^35^ In this study, the absorption of lutein did not affect the content of other carotenoids in animals. However, the study of Lakshminarayana et al. does not prove that lutein supplements can be directly incorporated into OO. The researchers incorporated powdered *C. benghalensis* (vegetable) material into OO that enriched the OO up to 2.69 mg lutein per kg OO as a formulation to feed to animals.

Considering the aforementioned evidence, OO, and especially extra virgin olive oil (EVOO) can be an important source of bioavailable dietary lutein. ^137^ Studies have shown that lutein loses a significant portion of its therapeutic value after several weeks of storage at room temperature. Montesano and colleagues, for example, showed that EVOO enriched with fruit-derived lutein underwent intensive auto-oxidation reactions over the 28 weeks of storage at room temperature. ^137^ Although the EVOO mixture did not increase beyond the acceptable limits of peroxide value (PV) that characterize an EVOO (20 meq O _2_/Kgr oil), ^138^ it had almost tripled its PV (>14 meq O _2_/Kgr oil) over that period of time. This rapid increase of PV predicts a reduction in its quality in terms of total polyphenols and intensive oxidation reactions carried out in the oil. ^139^

EVOO suffers from auto-oxidation reactions that degrade its valuable polyphenol content over time. ^140^ In this regard, another methodology of EVOO purification has important implications for lutein preservation. According to the molecularly filtered method applied to EVOO, ^141^ the content of lutein is preserved efficiently over a period of 24 months [Kyriakopoulos AM, personal communication and research currently accepted for publication]. The purified EVOO produced from this method has been shown to be beneficial for the topical treatment of psoriasis in human subjects. ^141^^,^^142^ In terms of long COVID and mRNA injury cases, EVOO constituents (poly-unsaturated fatty acids and polyphenols) can also prove beneficial and act synergistically with lutein.

Recent *in silico* studies predict that the two most valuable ingredients of EVOO, oleocanthal and oleuropein, can potentially inhibit the spike protein’s interaction with the ACE2 receptor. This inhibition has been demonstrated against the kappa, omicron and delta variants. ^143^ Therefore, these essential polyphenols must also be conserved in EVOO during the intake of lutein. It is suggested that EVOO rich in lutein must be consumed raw without mixing with other foods during intake, as was done in the animal studies. ^35^

## 9. Synergy with other natural compounds against long COVID and mRNA vaccination injuries

ACE2 binding by the spike protein RBD is the first step in virus infection, and therefore any agent that can disrupt this binding should have therapeutic value. ^144^ The recently discovered molecular docking properties of lutein on spike protein’s interface with ACE2 receptors ^52^ is therefore significant. To the extent that the SP/ACE2 interface is a key step in SP-associated pathologies, lutein’s strategic interference could help mitigate a wide range of post-COVID and mRNA vaccine injury sequelae. ^114^^,^^145^

In this regard, a related *in silico* docking study ^52^ points to lutein’s synergistic interaction with another phenolic compound, quercetin. The study predicts that, by binding to different spike protein structural locations, the combination of quercetin and lutein can enhance the destabilization of spike on the ACE2 interface. Moreover, it has been shown that quercetin exerts anti-COVID-19 activity by inhibiting the expression of ACE2 receptors. ^146^ Curcumin, another highly lipophilic phytochemical, was shown in an *in silico* study to also have potentially inhibitory properties against the spike protein of the new SARS-CoV-2 variants. ^147^ Curcumin can thus act synergistically with lutein. Quercetin, curcumin, and lutein all have anti-inflammatory properties that prompted this investigation into potential synergistic activities against post-COVID and mRNA vaccination injuries. Moreover, quercetin and curcumin have been considered as natural potential therapeutic agents against SARS-CoV-2 infection. ^146^^,^^148^ In addition, bromelain, another naturally derived potent anti-inflammatory and proteolytic molecule, can be used synergistically with the aforementioned compounds to enhance the anti-inflammatory response. ^149^ Finally, the fibrinolytic enzyme nattokinase can be used in conjunction with lutein, as it has recently been shown to effectively degrade the spike protein of SARS-CoV-2. ^150^

## 10. Conclusions

Abundant evidence suggests that lutein could be an effective natural agent for use against chronic illnesses and long COVID and mRNA injury syndrome. Both conditions share the same pathogenic factor that provokes the disease, which is the SARS-CoV-2 spike protein. Due to the enhanced antioxidant properties of the molecule over the other xanthophylls and carotenoids, lutein can offer the most effective oxidation homeostatic balance, restore normal levels of NO, and alleviate ROS/RNS imbalance. Particularly in long COVID and mRNA injuries, the levels of NO are altered, indicating nitrosative stress apart from oxidative stress. Although NO is not highly reactive, its derivatives generated with ROS reactions, such as peroxynitrate and N _2_O _3_, are highly reactive with larger biomolecules. Therefore, the impaired levels of NO can be a causative factor for arrhythmogenic cardiac failure. On these grounds, lutein’s adjuvant use for therapy has the potential to be lifesaving in these conditions. This is apart from lutein’s potential contribution to cardiovascular, kidney, liver, and neural-brain protection.

Furthermore, lutein can be useful against conditions of cognitive failure and dementia occurring from chronic inflammation in long COVID syndrome. Lutein’s antioxidant properties are complemented by its potential structural neutralization abilities against spike/ACE2 interactions. The spike protein is a long-lived circulating molecule in long COVID syndrome, and it is detected in autopsy cases of mRNA vaccination-induced cardiovascular complications that result in death. Therefore, lutein’s complementary docking abilities on the spike protein interface to the ACE2 receptor make it a molecule of high interest for preventing these conditions. A lutein-rich dietary intake is preferred over a high dose supplement, based on the contradictory results from eye-disease clinical studies using supplements.

EVOO can be the best carrier for intestinal absorption and accumulative dispersal of lutein in the tissues. In the way of natural supplementation, the competitive inhibition of other essential carotenoids (like β-carotene) absorption, accumulation, and activity can be avoided. Modern techniques on EVOO purification may naturally enhance the stability of derived lutein in EVOO. Complementarily, the valuable polyphenols and polyunsaturated fatty acids of EVOO can act synergistically with lutein to inhibit the spike protein and offer additional anti-inflammatory activities. Furthermore, lutein can be combined with the natural anti-inflammatory compounds such as curcumin, bromelain, quercetin and the proteolytic enzyme nattokinase to effectively inhibit the spike protein of SAR-CoV-2 and its variants from action. We conclude with a famous quote from Hippocrates: “Let food be thy medicine and medicine be thy food.” This may well be applicable in the case of lutein.

## Data Availability

No data are associated with this article.
