# Supplementary material for: Clinical rationale for dietary lutein supplementation in long COVID and mRNA vaccine injury syndromes
Source: F1000Res. 2024 Oct 17;13:191. Originally published 2024 Mar 15. [Version 3] doi: 10.12688/f1000research.143517.3 (PMC11549548; doi:10.12688/f1000research.143517.3)
Supplement: Peer review history

# Reviewer report: Reviewer response for version 3

**DOI:** 10.5256/f1000research.173439.r332741

**Published:** 2024-11-08

**Authors:** Andre Marolop Pangihutan Siahaan

**Affiliations:** 1 Universitas Sumatera Utara, North Sumatera, Indonesia

The authors have addressed all my previous comments.  I have no issue left.

Is the review written in accessible language?

Yes

Are all factual statements correct and adequately supported by citations?

Yes

Are the conclusions drawn appropriate in the context of the current research literature?

Yes

Is the topic of the review discussed comprehensively in the context of the current literature?

Yes

Reviewer Expertise:

Evidence based medicine.

I confirm that I have read this submission and believe that I have an appropriate level of expertise to confirm that it is of an acceptable scientific standard.

# Reviewer report: Reviewer response for version 2

**DOI:** 10.5256/f1000research.169744.r324401

**Published:** 2024-10-10

**Authors:** Andre Marolop Pangihutan Siahaan

**Affiliations:** 1 Universitas Sumatera Utara, North Sumatera, Indonesia

The author conducted a well-written review. I only had several issues.

  

1. Based on the pharmacokinetics of lutein distribution, typically, circulating carotenoids are transported primarily through LDL (55%), HDL (33%), VLDL (10-19%), with recent study stating a significant positive correlation between lipoprotein concentrations and serum levels of lutein dan zeaxanthin and concluding that altering lipoprotein concentrations may affect the levels of retinal lutein and zeaxanthine. Moreover, lutein, due to its lipophilic nature is more commonly distributed in adipose tissues. Consequently, obese people have lower lutein level, exposing them to the higher risk of various eye diseases.  Due to that, lipoprotein levels should be included as a variable in this study to furthermore explore the possibilities of lutein as a systemic antioxidant.
2. Studies showed a negative correlation between changes in adipose tissue lutein concentration and changes in MPOD after stopping lutein supplementation. It would also be reasonable to question how long the effects of lutein would stay after its cessation in the case of reducing inflammations in patients with Long COVID or Post-Vaccination Syndrome and to consider the period a person needs to receive lutein supplementation to receive significant benefits.

Congratulations on the excellent work.

Is the review written in accessible language?

Yes

Are all factual statements correct and adequately supported by citations?

Yes

Are the conclusions drawn appropriate in the context of the current research literature?

Yes

Is the topic of the review discussed comprehensively in the context of the current literature?

Yes

Reviewer Expertise:

Evidence based medicine.

I confirm that I have read this submission and believe that I have an appropriate level of expertise to confirm that it is of an acceptable scientific standard, however I have significant reservations, as outlined above.

# Reviewer report: Reviewer response for version 2

**DOI:** 10.5256/f1000research.169744.r317253

**Published:** 2024-09-04

**Authors:** Konstantinos Poulas

**Affiliations:** 1 Department of Pharmacy, University of Patras, Patras, Greece

Authors have responded to the requests properly. It can be indexed  after these modifications.

Is the review written in accessible language?

Yes

Are all factual statements correct and adequately supported by citations?

Yes

Are the conclusions drawn appropriate in the context of the current research literature?

Yes

Is the topic of the review discussed comprehensively in the context of the current literature?

Yes

Reviewer Expertise:

Immunology

I confirm that I have read this submission and believe that I have an appropriate level of expertise to confirm that it is of an acceptable scientific standard.

# Response

**Published:** 2024-09-04

**Authors:** Stephanie Seneff

**Affiliations:** CSAIL, Massachusetts Institute of Technology, Cambridge, Massachusetts, USA

Wonderful news. Thank you for your efforts to improve this paper.

# Reviewer report: Reviewer response for version 1

**DOI:** 10.5256/f1000research.157190.r288979

**Published:** 2024-07-01

**Authors:** Konstantinos Poulas

**Affiliations:** 1 Department of Pharmacy, University of Patras, Patras, Greece

Very detailed and important manuscript by Kyriakopoulos et al, about the well established role of lutein as antioxidant and anti-inflammatory factor for a great number of pathologies.

 The authors, apart from reviewing the current literature, are proposing the beneficial effect of lutein for post-COVID and post-vaccination health implications.

 I don't have any major comment. Knowing, however, that there is a debate about the existence or not of the so-called post-COVID syndrome, I would like the authors to include and discuss this debate in the manuscript, with the appropriate references.

 They present, Figure 2, that post-COVID and post-vaccination pathologies are presenting different/controversial pathophysiological pathways. Since there is a strong possibility that post-COVID is a non-existent pathology, I would like the authors to modify the manuscript, wherever they present post-COVID pathophysiological characteristics in comparison with the post-vaccination pathophysiological pathways.

Is the review written in accessible language?

Yes

Are all factual statements correct and adequately supported by citations?

Yes

Are the conclusions drawn appropriate in the context of the current research literature?

Yes

Is the topic of the review discussed comprehensively in the context of the current literature?

Yes

Reviewer Expertise:

Immunology

I confirm that I have read this submission and believe that I have an appropriate level of expertise to confirm that it is of an acceptable scientific standard, however I have significant reservations, as outlined above.

# Response

**Published:** 2024-07-29

**Authors:** Stephanie Seneff

**Affiliations:** CSAIL, Massachusetts Institute of Technology, Cambridge, Massachusetts, USA

**Response to Reviewer Comments**

 We understand the reviewer’s concerns about post COVID and mRNA related injuries.

 Therefore, we have made the following changes to the manuscript.

1. Since long COVID is a well-recognised syndrome, we have changed “post COVID (syndrome)” to “long COVID” throughout the text.
2. We have inserted a new Section 2., explaining that long COVID and mRNA injury syndrome disease manifestations have a common origin. This is the SARS-CoV-2 spike protein induced pathology. Nowadays, most of the patients presenting with long term spike protein induced pathology are mRNA vaccinated, whereas in the unvaccinated population the long-term spike pathologies are more modest and infrequent. We address this issue by inserting a new reference: https://pubmed.ncbi.nlm.nih.gov/37634619/. In this study, it is quoted that long term COVID complications are more likely to emerge in the mRNA vaccinated individuals. In the group of individuals having a previous SARS CoV-2 infection (presumably not vaccinated) the risk factor for having long COVID complications does not increase.
3. In Table 2, since most of the studies included are at the beginning of the COVID-19 pandemic, we assume that the patient results included are mainly from individuals who have not been vaccinated. Otherwise, the authors of these studies would have mentioned mRNA vaccination for the patients they investigated.
4. In the conclusion section, we have inserted a specific sentence stating that long COVID and mRNA vaccination injury syndromes share the same origin of pathology, the SARS CoV-2 spike protein. 

Since the scope of this study is to investigate the potential therapeutic role of lutein against the SARS CoV-2 spike protein induced pathology, we consider that these above changes are enough to cover the scientifically well based reviewer comments.
